# An Efficient Ship Detection Method Based on YOLO and Ship Wakes Using High-Resolution Optical Jilin1 Satellite Imagery

**DOI:** 10.3390/s24206708

**Published:** 2024-10-18

**Authors:** Fangli Mou, Zide Fan, Yunping Ge, Lei Wang, Xinming Li

**Affiliations:** Key Laboratory of Target Cognition and Application Technology, Aerospace Information Research Institute, Chinese Academy of Sciences, Beijing 100190, China; mfl_aircas@hotmail.com (F.M.); geyp@aircas.ac.cn (Y.G.); wanglei002931@aircas.ac.cn (L.W.); 13911729321@139.com (X.L.)

**Keywords:** maritime surveillance, vessel detection and tracking, wake detection, marine engineering, remote sensing processing

## Abstract

In this study, we propose a practical and efficient scheme for ship detection in remote sensing imagery. Our method is developed using both ship body detection and ship wake detection and combines deep learning and feature-based image processing. A deep convolutional neural network is used to achieve ship body detection, and a feature-based processing method is proposed to detect ship wakes. For better analysis, we model the sea region and evaluate the quality of the image. Generally, the wake detection result is used to assist ship detection and obtain the sailing direction. Conventional methods cannot detect ships that are covered by clouds or outside the image boundary. The method proposed in this paper uses the wake to detect such ships, with a certain level of confidence and low false alarm probability in detection. Practical aspects such as the method’s applicability and time efficiency are considered in our method for real applications. We demonstrate the effectiveness of our method in a real remote sensing dataset. The results show that over 93.5% of ships and over 70% of targets with no visible ship body can be successfully detected. This illustrates that the proposed detection framework can fill the gap regarding the detection of sailing ships in a remote sensing image.

## 1. Introduction

The capability to automatically detect sailing ships has a wide range of applications in the marine and commercial fields, such as ensuring the safety of sailing routes, performing maritime supervision, and combating pirates [1]. Remote sensing has a wide view with a high resolution, and it plays an irreplaceable role in ocean surveillance [2], especially when the target’s automatic identification system (AIS) is disabled. An AIS is a system that can track and report the sailing status of ships; it is influenced by the equipment quality and protocol standards and can be turned off artificially.

According to the type of sensor, remote sensing imagery can be divided into two categories: synthetic aperture radar (SAR) images and visible images. Different from passive sensors, such as optical and infrared sensors, SAR actively emits microwaves for observation, and it is not subject to climatic factors such as light, temperature, and clouds. These advantages mean that SAR can carry out Earth exploration operations during the day and night with long-term stability, and it has a wide range of applications in the field of ocean exploration. Researchers have proposed many ship detection methods based on SAR images [3,4,5]. Generally, ship detection methods using SAR images can be divided into direct detection methods and indirect detection methods. A direct detection method uses the pixel difference between ships and the sea background to detect ships; these methods are mainly based on the statistical features of SAR images [6], and the constant false alarm rate (CFAR) method is the most representative direct detection method [7]. The CFAR method analyzes the statistical characteristics of the sea background and creates a distribution model to judge whether the current pixel is the target pixel according to the threshold obtained from the given false alarm rate. The distribution model of the sea background [8,9,10] and the detector design method [11,12,13] are the most important aspects in CFAR; the image segmentation method is also used to improve the detection performance in SAR images [14]. Indirect detection methods aim to detect ships by detecting auxiliary features (such as ship wakes). When the features of the ship body in an image are not obvious, the detection of auxiliary features is particularly important. The wakes caused by ship sailing can last for a considerable period of time over a large area on the sea surface [15]. Therefore, the observation probability of ship wakes is larger than that of the ship body, and the detection characteristics are more obvious than those of the target itself. In addition, by combining the wake with the position of the target in the image, the speed, the course, and the actual position of the target can be predicted more accurately. Ship wakes usually have linear features in SAR images [16], and traditional methods use line detection methods to achieve wake detection. The Hough transform [17] and Radon transform [18] are the most common line detection algorithms and are widely used in wake detection [19,20,21,22,23,24,25]. In recent years, learning-based methods have also been proposed to achieve this and show great potential in ship detection in SAR images [26,27,28]. These methods require training and labeled datasets, and there is no presented ship wake dataset for training, which makes wake detection difficult to perform.

Compared with SAR, visible imagery has the advantages of visualization and a low cost; moreover, visible remote sensing imagery has a high resolution and can obtain more local detail features. Thus, the results are more convenient for understanding and further analysis. Conventional detection methods are usually based on visual saliency and visual perception principles, and the Itti model [29], Ft model [30], SR model [31], AC model [32], and GBVS model [33] are widely used in extracting ship regions from the sea background. Moreover, the Histogram of Oriented Gradients (HOG) descriptor is a commonly used feature descriptor in ship detection [34]. In general, the above traditional algorithms have unsatisfactory detection performance under complex background environments. The convolutional neural network (CNN)-based method is especially effective in ship detection in visible remote sensing images. Deep learning methods can effectively overcome the shortcomings of human design, and, due to the large capacity of the model, the target can be described in detail, which greatly improves the accuracy of detection. According to their processing steps, deep learning networks can be divided into two-stage detection methods and single-stage detection methods. A two-stage detection method is carried out with the structure of traditional methods. The whole image is traversed to generate several candidate boxes, and then the candidate boxes are classified to determine whether there is a target in the area; then, the network is used to finely locate and classify the target. R-CNN [35], Fast R-CNN [36], and Mask R-CNN [37] are the most commonly used frameworks. The single-stage detection method was proposed to predict multiple bounding boxes and obtain class probabilities for these boxes simultaneously in one evaluation. You only look once (YOLO) [38,39] is one of the most representative methods that can perform real-time detection with fewer false positive detections on the background.

These proposed methods focus on the detection of the ship body, and there are few reports on the study of ship wakes in visible remote sensing images. As mentioned, visible remote sensing imagery is influenced by climatic factors, and wakes can be used to assist ship detection. However, in high-resolution visible remote sensing images, the ship wakes have obvious local details and are not visually represented as a fine line segment but as a region with a certain shape. This means that the traditional methods used for SAR images are difficult to apply to visible remote sensing images. The spatial resolution is the key to detecting objects using optical satellite images; however, the spatial resolution of an image is sometimes unavailable, and image preprocessing is often a necessary step. Hence, it is helpful to evaluate the image quality, which is independent of the spatial resolution, to guide the algorithm design. Moreover, the proposed method is suitable for a target vessel occupying a certain region; the width of the target vessel should be over five pixels. This method is influenced by the size of the target vessel and the spatial resolution of the satellite image.

This study addresses the ship detection task for visible remote sensing images. We present a general detection scheme combining deep learning and feature-based image processing. We fuse the detection results of the ship body and ship wakes to achieve robust ship detection. A YOLOv5 [40] network is used to achieve the effective detection of the ship body, and a feature-based image processing method is proposed for wake detection. The sea region is modeled and the quality of the image is evaluated. The wake detection result is used to assist ship detection and obtain the sailing direction. Using wake detection, invisible ships that are covered by clouds or are outside the image boundary can also be detected with a certain level of confidence. We demonstrate the effectiveness of our method in a real dataset, which shows that the proposed detection framework can fill the gap in the detection of sailing ships in a remote sensing image. Moreover, the method’s performance, varying with the proposed criterion, is also discussed.

As far as the authors are concerned, this is the first attempt to fuse ship body detection and ship wake detection to solve the problem of invisible vessel detection in real applications.

In brief, the contributions of this study can be summarized as follows.

1. We propose an effective and practical ship detection method for visible remote sensing images, which takes full advantage of deep learning and feature-based image processing.
2. We propose a useful assessment method to analyze the quality of visible remote sensing images, which can calculate the confidence of the detection results.
3. The proposed method can achieve detection even when the targets are invisible in a certain image, which is difficult to realize with the current methods.

The remainder of this paper is organized as follows. Section 2 presents the problem definition and statement. Section 3 describes our ship detection method based on YOLO and ship wakes. The detection results are presented and discussed in Section 4. Finally, conclusions are drawn and future work is discussed in Section 5.

## 2. Problem Statement

**Figure 1:**
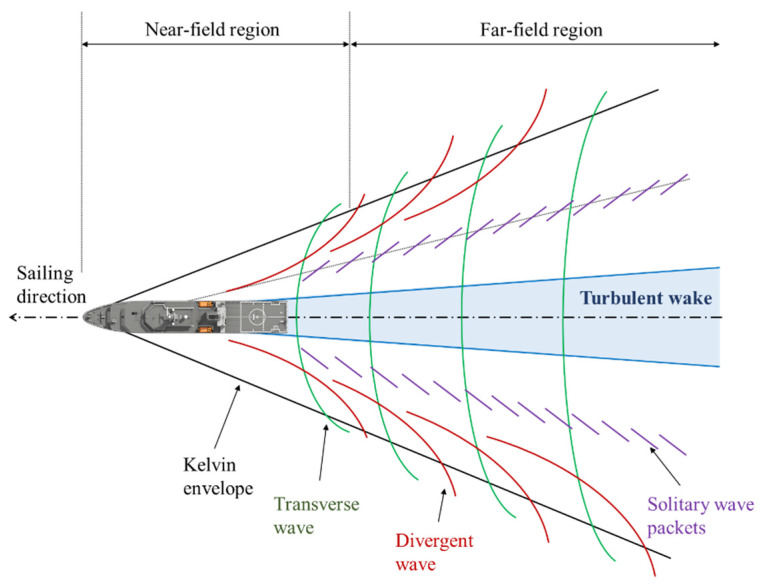
Illustraction of ship wake patterns.

In this study, we focus on ships sailing on the ocean; the ship wakes are the most significant characteristic for researchers seeking to analyze the ship state. The wakes can remain for a long time at the sea surface and can be used to estimate the sailing direction. Sailing ships produce four categories of wake structures; these are the Kelvin wake, the turbulent wake, the narrow V wake, and the internal wave wake [41]. These classic wake structures are shown in Figure 1.

**Figure 2:**
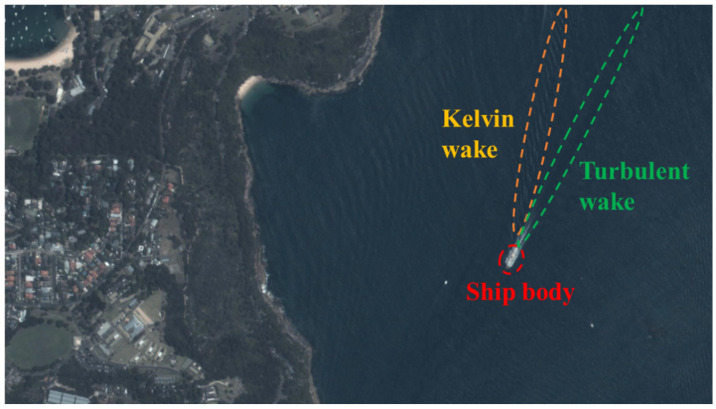
Ship wakes in remote sensing image.

The ship wakes in a remote sensing image are shown in Figure 2. We can observe that the ship wakes in a visible image are not as pronounced as those in an SAR image, and the ship body has more contrast with the sea background. This indicates that wake detection is more difficult than ship detection in a remote sensing image. However, direct ship detection also suffers from problems due to cloud coverage and detection around the image boundary, and, in these cases, the long-term remaining wakes can be used for direct ship detection.

**Figure 3:**
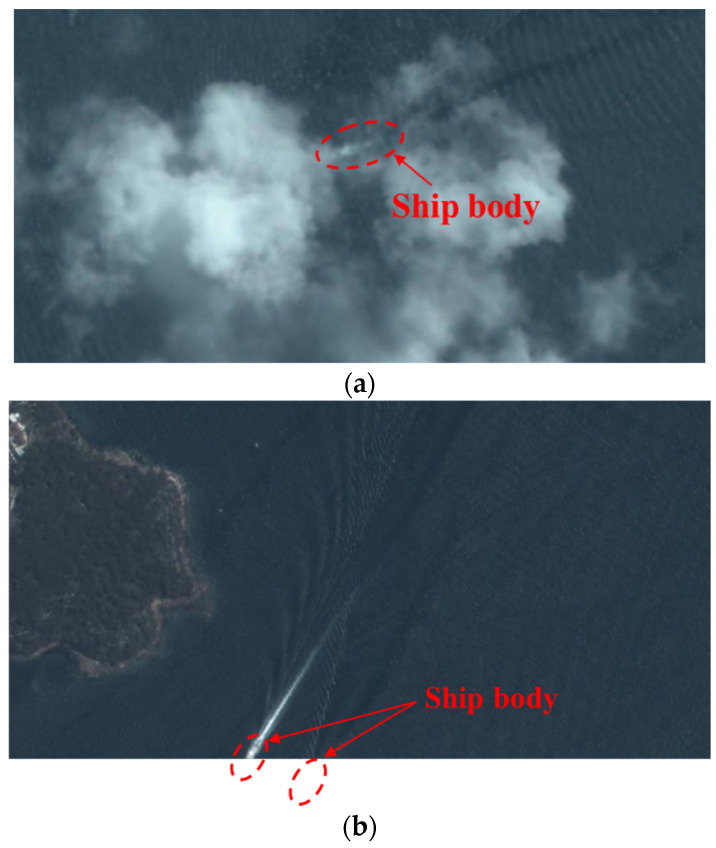
Detection gaps in remote sensing image: (**a**) cloud coverage; (**b**) detection around image boundary.

Currently, the average revisit time of satellites is large (about 20 min) and the sensing cost is high. Using more information from remote sensing images can significantly increase the efficiency of marine observation. This is the core idea of this paper, using indirect wake detection to fill the detection gap regarding sailing ships in a remote sensing image, as shown in Figure 3.

## 3. Proposed Ship Detection Method

**Figure 4:**
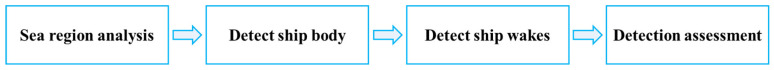
General ship detection process in this paper.

**Figure 5:**
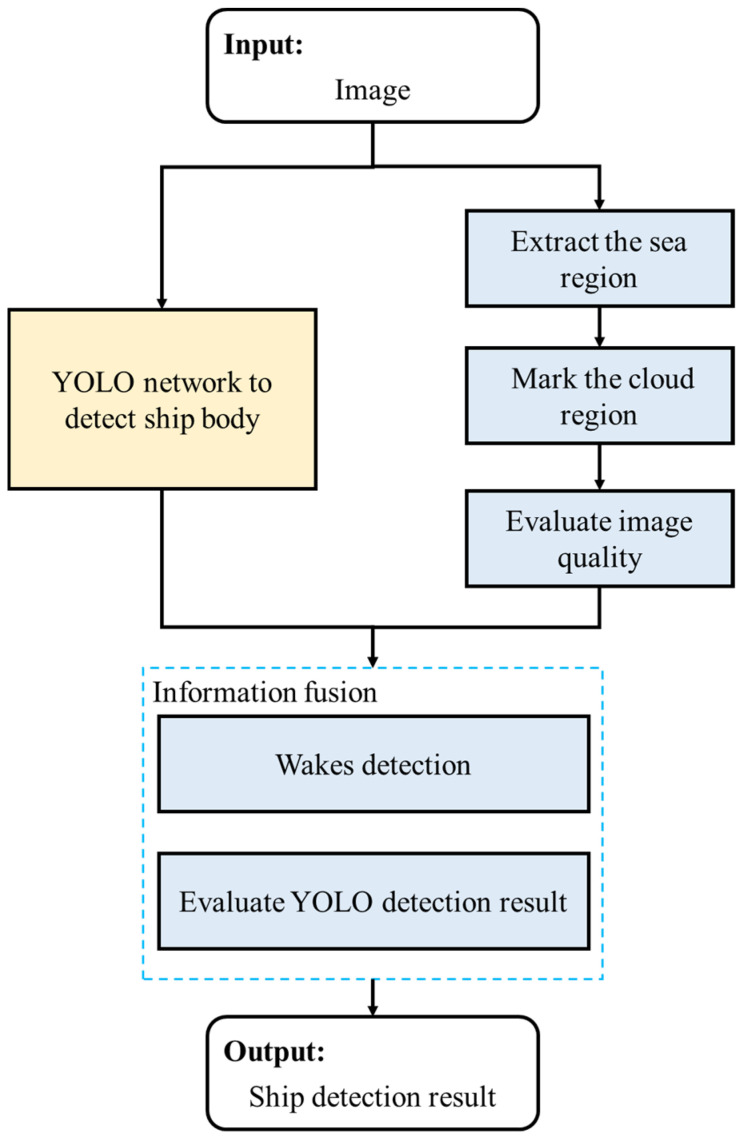
General flowchart of our method.

The general ship detection process is depicted in Figure 4. Sea region analysis extracts the sea area in a remote sensing image and evaluates the image quality, which limits the detection results. Then, we sequentially perform ship body detection and ship wake detection and evaluate the detection result. We develop our method based on the necessary requirements, the limited computing resources, and the current research status. The general flowchart of our method is shown in Figure 5. As mentioned, we combine ship body detection and the information of ship wakes to achieve ship detection. The used YOLO network presents efficient detection for the ship body; the fusion framework and the usage of wakes are the main focus in this paper. We note that our method can be regarded as an additional step after the conventional detection network.

### 3.1. Sea Region Analysis

When we obtain a new image, we first use a Gauss filter with small variance to denoise the original remote sensing image. The sea region analysis consists of the following steps.

**Step 1: Extract the sea region.**

In a visible image, the sea region $C_{sea}$ usually has a color close to blue, so we first convert the image from the RGB color space to the LAB color space in order to have a more effective color comparison. We use several manually segmented images to compute the mean value of the sea region, which is represented by the A value and B value.

When given an image, we perform k-means++ clustering [42] in the AB subspace, using the mean value of the sea region as the initial cluster centroid. To improve the efficiency, we only choose two cluster centroids to represent whether the pixel belongs to the ocean region or not.

After clustering, we perform morphological opening and closing on the extracted ocean region to remove snowflakes and fill the holes. We mark the region of interest according to the result of the connected component analysis.

**Step 2: Mark the cloud region in the sea region.**

Cloud coverage causes the invisibility of the target in a remote sensing image. We mark the cloud region $C_{cloud}$ based on the dark channel prior (DCP) [43]. The dark channel of an image can be given by

(1) $$J^{dark}(x)=\underset{c\in \{r,g,b\}}{\min}(\underset{y\in \Omega (x)}{\min}J^{c}(y))$$

Here, $J^{c}$ is a color channel of the remote sensing image and Ω(x) is a local patch centered at x. Excluding the cloud region, the dark channel of the image has a value close to zero.

We mark the first 0.1% of the pixels from the dark channel image in order of brightness as the cloud pixels and then successively perform morphological opening, morphological closing, and connected component analysis to obtain the cloud region. The DCP method does not rely on significant variance in transmission or surface shading; however, the calculation is performed using global and local statistical information, and illumination correction is needed to provide relatively uniform correction.

**Step 3: Evaluate the quality of the image.**

**Figure 6:**
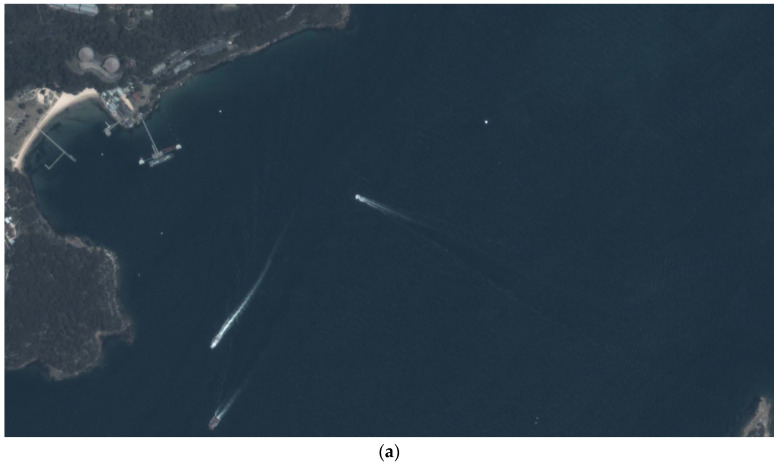

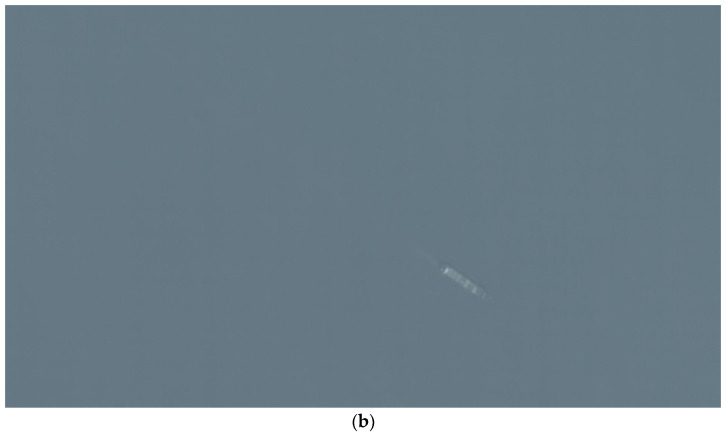
Observability caused by image quality (**a**,**b**).

We note that the quality of the image has a significant influence on the analysis of the image features. In Figure 6, we can observe the waves and wakes caused by the vessel in Figure 6a; however, these features are extremely faint in Figure 6b. Due to the aim of this paper, we only focus on the ocean part in the image. The ocean background $C_{bg}$ is the difference set of the ocean region $C_{sea}$ and cloud region $C_{cloud}$:

(2) $$C_{bg}=C_{sea}-C_{cloud}$$

For the quantitative analysis, we propose the following quality criterion to describe the quality of an image:

(3) $$QI=\prod_{i=1}^{n}I_{i}^{\frac{w_{i}}{n}},QI\in [0,1]$$

Generally, a larger QI reflects a higher spatial resolution. A larger value of QI indicates better image quality. Here, $I_{i}\in [0,1]$ represents the normalized indicator generated by a characteristic of the image, and $w_{i}$ is the weight of the corresponding indicator. Four characteristics are used to calculate the image quality in this paper; they are the image entropy $H_{e}$ [44], Tenengrad gradient $H_{T}$ [45], Gabor feature $H_{G}$, and reblur performance $H_{R}$.

The image entropy $H_{e}$ is computed as

(4) $$H_{e}=-\sum \left(p\times log_{2}(p)\right)$$

where p contains the normalized histogram count of the image, and a larger value of $H_{e}$ indicates better image quality.

The Tenengrad gradient $H_{T}$ is computed as

(5) $$H_{T}=\sum (G_{x}^{2}(x,y)+G_{y}^{2}(x,y))$$

where $G_{x}(x,y),G_{y}(x,y)$ represent the directional gradient in the *x* and *y* directions, respectively, and a larger value of $H_{T}$ indicates better image quality.

The Gabor filter is a is a linear bandpass filter that is widely used in image processing for edge detection, texture classification, feature extraction, and disparity estimation [46]. The Gabor feature $H_{G}$ is the maximum image entropy of the response matrix $\left\{R_{G}\right\}$ obtained by convolving the original image $Im_{0}$ with the Gabor filters {g(x,y|λ,θ,ψ,σ,γ)}:

(6) $$\begin{array}{l} g(x,y|\lambda ,\theta ,\psi ,\sigma ,\gamma )=\exp(-\frac{{\tilde{x}}^{2}+\gamma^{2}{\tilde{y}}^{2}}{2\sigma^{2}})\exp\left[i\left(2\pi \frac{\tilde{x}}{\lambda }+\psi \right)\right],\\ \tilde{x}=x\cos\theta +y\sin\theta ,\tilde{y}=-x\sin\theta +y\cos\theta \end{array}$$

and

(7) $$\begin{array}{l} R_{G}=g(x,y|\lambda ,\theta ,\psi ,\sigma ,\gamma )*Im_{0}\\ H_{G}=\max(H_{e}(\{R_{G}\})) \end{array}$$

Here, we choose orientations $\theta =[0,\frac{\pi }{6},\frac{\pi }{3},\frac{\pi }{2},\frac{2\pi }{3},\frac{5\pi }{6}]$ and wavelength λ=[2,4,8], and a larger value of $H_{G}$ indicates better image quality.

The reblur operation is based on the idea that the blurred image loses less information after smoothing [47]. The reblur performance $H_{R}$ reflects the similarity between the original image $Im_{0}$ and the blurred image $Im^{\prime }$:

(8) $$H_{R}=SSIM(Im^{\prime },Im_{0})$$

Here, $Im^{\prime }=G_{f}(\sigma )*Im_{0}$ is the blurred image using a Gaussian filter $G_{f}$ with variance of σ; SSIM() is the Structure Similarity Index Measure (SSIM), used to measure the similarity between two images from the same capture, which is based on the idea that the human visual system is sensitive to structural information in a scene [48]:

(9) $$\begin{array}{l} SSIM(Im^{\prime },Im_{0})={\left[l(Im^{\prime },Im_{0})\right]}^{\alpha }\cdot {\left[c(Im^{\prime },Im_{0})\right]}^{\beta }\cdot {\left[s(Im^{\prime },Im_{0})\right]}^{\gamma }\\ l(Im^{\prime },Im_{0})=\frac{2\mu_{x}\mu_{y}+C_{1}}{\mu_{x}^{2}+\mu_{y}^{2}+C_{1}},c(Im^{\prime },Im_{0})=\frac{2\sigma_{x}\sigma_{y}+C_{2}}{\sigma_{x}^{2}+\sigma_{y}^{2}+C_{2}},s(Im^{\prime },Im_{0})=\frac{\sigma_{xy}+C_{3}}{\sigma_{x}\sigma_{y}+C_{3}} \end{array}$$

where $\mu_{x},\mu_{y},\sigma_{x},\sigma_{y},\sigma_{xy}$ are the local means, standard deviations, and cross-covariance for images $Im_{0}$ and $Im^{\prime }$; $C_{1},C_{2},C_{3}$ are the small regularization constants; α,β,γ are the weighting coefficients. A value of $H_{R}$ closer to 0 indicates better image quality.

These indicators $I_{i}$ are calculated as

(10) $$\begin{array}{l} I_{1}={\left(1+\exp(a_{1}(H_{e}-b_{1}))\right)}^{-1},I_{2}={\left(1+\exp(a_{2}(H_{T}-b_{2}))\right)}^{-1}\\ I_{3}={\left(1+\exp(a_{3}(H_{G}-b_{3}))\right)}^{-1},I_{4}=1-{\left(1+\exp(a_{4}(H_{R}-b_{4}))\right)}^{-1} \end{array}$$

where $a_{1},a_{2},a_{3},a_{4}$ and $b_{1},b_{2},b_{3},b_{4}$ are the parameters.

The performance of these indicators can be seen in the performance of image quality indicators in Section 4.3. According to the QI, we can determine the subsequent operation procedure and the general confidence of detection.

These indicators are relatively robust in practical scenarios. Under extreme weather conditions (e.g., strong winds, high waves), QI may not be greatly influenced. However, in this case, the false alarms during detection may increase, since wakes can be greatly influenced by extreme weather conditions. For a sea state below moderate (usually 4–8 ft (1.25–2.50 m)), the proposed QI can have good robustness. If these challenging environments occur in an application, QI can be adapted in the following form:

$$QI=SI\times \prod_{i=1}^{n}I_{i}^{\frac{w_{i}}{n}},QI\in [0,1]$$

where SI is the sea state coefficient; for a sea state below moderate, SI=1, and, for a sea state above moderate, SI=0.

**Step 4: Model the ocean background.**

Here, we convert the ocean background to standardized grayscale and use the Burr type XII distribution [49] to model the ocean background, since we find that the background distribution has a unimodal form and is skewed to the right (as seen in the performance of cloud detection in Section 4.2). The cumulative distribution function (cdf) and probability density function (pdf) of the Burr distribution are

(11) $$\begin{array}{l} F(x|\alpha ,c,k)=1-\frac{1}{{\left(1+{\left(x/\alpha \right)}^{c}\right)}^{k}}\\ f(x|\alpha ,c,k)=\frac{kc}{\alpha }{\left(\frac{x}{\alpha }\right)}^{c-1}{\left(1+\frac{x}{\alpha }\right)}^{-k-1} \end{array}$$

where α,c,k>0 are the distribution parameters.

### 3.2. Detection of Ship Body

The ship body detection task aims to detect instances of ships with a certain characteristic. It includes a localization operation and a classification operation. Generally, the ship region $Ar_{ship}$ occupies a small space in a remote sensing image. For example, the used images usually have a size of 1920 × 1080, and the ship usually occupies a small space with a size of less than 200 × 200. These small objects mean that the traditional methods yield less satisfactory results. In recent years, deep learning has shown great potential in image processing and is widely used in object detection. YOLO is a one-stage real-time object detection approach that uses a single network to realize the entire detection pipeline. We use the dataset from [50] with image enhancement to train a YOLOv5 network [22] with one category to achieve the detection of the ship body. We find that the YOLOv5, YOLOv8, and YOLOX networks have similar performance in detecting sailing ships (all with over 90% detection accuracy, and the accuracy improvement less than 3%). Since we need to deploy our system in a certain equipment setup with limit computing resources, we finally choose the YOLOv5 network. We note that the used network is not the main concern of this paper; we simply use it as a tool to present regular ship body detection.

We further check the YOLO detection region, and we relax the detection region to two times the side length and keep only the relaxed regions in which over 50% of the area belongs to the ocean background $C_{bg}$. The accuracy of ship body detection can be over 90%.

### 3.3. Detection of Ship Wakes

We now describe our wake detection method, as well as its scope of application. Generally, the wake detection method consists of the following steps. We note that only images with a quality value QI larger than the distinct threshold $Th_{0}$ are considered meaningful to extract the wakes.

**Step 1: Determine the wake searching region.**

According to the purpose of usage, the wake searching region $S_{r}$ consists of three types: the ship body neighborhood $S_{1}$, the cloud neighborhood $S_{2}$, and the boundary region $S_{3}$.

The ship body neighborhood $S_{1}$ lies along the axis of the vessel detection window; since the ship a large length–width ratio, we use principal component analysis (PCA) to obtain the ship axis:

(12) $$\begin{array}{l} S_{\mathrm{cov}}=\frac{1}{n-1}\sum_{i=1}^{n}(x_{i}-\mu ){\left(x_{i}-\mu \right)}^{T}\\ S_{\mathrm{cov}}=\sum_{i=1}^{d}\lambda_{i}v_{i}v_{i}^{T}≃\sum_{i=1}^{k}\lambda_{i}v_{i}v_{i}^{T} \end{array}$$

Here, we binarize the vessel area and $S_{\mathrm{cov}}$ is the covariance matrix of the position matrix of the vessel area; $\lambda_{i},v_{i}$ are the eigenvalues arranged from large to small and the corresponding eigenvectors; for a two-dimensional image, k=d=2; and $v_{1},v_{2}$ are the long-axis direction and short-axis direction of the vessel, respectively.

$S_{1}$ is generated according to the minimum bounding rectangle of the binarized vessel area with a length of four times. The cloud neighborhood $S_{2}$ is generated according to the minimum bounding rectangle of the cloud region $C_{cloud}$ with a spacing distance of about 50~100 m. The boundary region $S_{3}$ also has margins of about 50~100 m.

**Step 2: Search the turbulent wake in** $S_{r}$**.**

Among the presented wake patterns in Figure 1, the Kelvin wake and the turbulent wake are the most likely wake patterns that will be observed in a remote sensing image. Compared with the Kelvin wake, the turbulent wake usually has a larger region and its grayscale has a more concentrated distribution. These make the detection of turbulent wakes more robust; hence, we detect the turbulent wake to aid ship detection in this paper.

For each wake searching region obtained in Step 1, we first convert the image from the RGB space to the LAB space. Since the L component directly reflects the brightness, which is a significant feature in distinguishing turbulent wakes, we also use the Burr type XII distribution to model the L component of the searching region. The log-likelihood function of the Burr type XII distribution is

(13) $$\log(L_{B})=-(k+1)\sum_{i=1}^{n}\log\left({\left(\frac{x_{i}}{\alpha }\right)}^{c}+1\right)+(c-1)\sum_{i=1}^{n}\log(x_{i})-cn\log(\alpha )+n\log(c)+n\log(k)$$

We use the maximum likelihood estimation (MLE) method to obtain the parameters. However, finite maximum likelihood estimates for the Burr type XII distribution do not always exist due to the possibility of having non-degenerated limiting distributions [51]. Here, we use the Gamma distribution as the alternative distribution form when the Burr type XII distribution cannot be solved. The pdf and cdf of the Gamma distribution are

(14) $$f_{G}(x|L,\mu )={\left(\frac{L}{\mu }\right)}^{L}\frac{x^{L-1}e^{-Lx/\mu }}{\Gamma (L)},F_{G}(x|L,\mu )=\frac{1}{\Gamma (L)}\int_{0}^{Lx/\mu }t^{L-1}e^{-t}dt$$

where x,L,μ>0, and the Gamma function Γ(a) is $\Gamma (a)=\int_{0}^{+\infty }t^{a-1}e^{t}dt$.

The MLE of the Gamma distribution parameters is

(15) $$\hat{\mu }=\frac{1}{n}\sum_{i=1}^{n}x_{i},\frac{d(\ln(\Gamma (L)))}{dL}=\ln\left(\frac{L}{\hat{\mu }}\right)+\frac{1}{n}\sum_{i=1}^{n}\ln x_{i}$$

Before we fit the distribution, we first standardize the L component x to obtain better fitting:

(16) $$\tilde{x}=x-\min(x)+\varepsilon$$

where ε is a small regularization constant.

Then, the bright turbulent wake and dark turbulent wake can be extracted based on the idea of CFAR:

(17) $$\begin{array}{l} \mathrm{For}\;\mathrm{Burr}\;\mathrm{type}\;\mathrm{XII}\;\mathrm{distribution}:\\ p_{1}=1-\frac{1}{{\left(1+{\left(T_{b}/\alpha \right)}^{c}\right)}^{k}},p_{2}=\frac{1}{{\left(1+{\left(T_{d}/\alpha \right)}^{c}\right)}^{k}}\\ \mathrm{For}\;\mathrm{Gamma}\;\mathrm{distribution}:\\ p_{1}=1-{\left(\frac{L}{\mu }\right)}^{L}\int_{0}^{T_{b}}\frac{1}{\Gamma (L)}t^{L-1}e^{-Lt/\mu }dt,p_{2}={\left(\frac{L}{\mu }\right)}^{L}\int_{0}^{T_{d}}\frac{1}{\Gamma (L)}t^{L-1}e^{-Lt/\mu }dt \end{array}$$

where $p_{1}$ and $p_{2}$ are the given region probabilities of bright and dark turbulent wakes, respectively; $T_{b}$ and $T_{d}$ are the calculated detection thresholds.

The bright turbulent wake region $W_{b}$ and dark turbulent wake region $W_{d}$ can be obtained by

(18) $$W_{b}=\{x_{L}:x_{L}\geq T_{b}\},W_{b}=\{x_{L}:x_{L}\leq T_{d}\}$$

Since noise waves may also be extracted as ship wakes, we use the density-based spatial clustering of application with noise (DBSCAN) method [52] to cluster the wakes.

The DBSCAN algorithm constructs the ε-neighborhood of the data point as

(19) $$N_{\varepsilon }(p)=\{q\in X^{c}|dist(p,q)\leq \varepsilon \}$$

where dist is the distance function; we choose the $L_{1}$ norm as the distance function in our method.

The DBSCAN method uses the neighborhood density threshold $M_{\varepsilon }$ to discover the clusters of the dataset that contain at least $M_{\varepsilon }$ central points. These parameters can be chosen according to the image resolution, and, in our case, they can be chosen as

(20) $$\varepsilon =15,\;M_{\varepsilon }=5$$

The performance of the DBSCAN method can be affected by these parameters. In our case, the spatial resolution of these images is from 0.91 m to 1.27 m, and the chosen parameters are robust in this case. Generally, when the spatial resolution increases, the parameter $M_{\varepsilon }$ increases while parameter ε decreases. Since the wakes have a larger region in images with high quality and a high spatial resolution, a more adaptive approach can be used via a data-driven method. The NN-based method can be used when we have a large number of data and the RBF-based method can be used when we have fewer data.

Since the wake has a banded spatial distribution, we use the bivariate normal distribution Ν(μ,Σ) to model the wake region. The covariance matrix Σ reflects the shape of the extracted region; we use the ellipticity of the equiprobability curve as the criterion $J_{W}$ to check the wake region obtained by DBSCAN:

(21) $$J_{W}=1-\sqrt{\frac{\lambda_{\min}}{\lambda_{\max}}}$$

where

(22) $$\Sigma^{-1}=U\Lambda U^{T},\Lambda =\left[\begin{array}{cc} \lambda_{\max} & \\ & \lambda_{\min} \end{array}\right]$$

Only the region with an area larger than $A_{0}$ and where $J_{W}>J_{0}$ is considered as the wake region. Next, we use a quadratic curve to fit each wake region and merge these regions with a similar fitting curve.

We note that the proposed method of wake extraction relies on the image quality and spatial resolution. Only for images with a high QI (QI>0.7) can the wakes be extracted using the proposed method, and it shows a good improvement when QI>0.85. Since the wakes are faint in low-spatial-resolution or low-quality images, no additional information can be acquired using the proposed method. The detailed information can be seen in the analysis in Section 4.4.

### 3.4. Detection Assessment

After the above process, we use the following principle to guide ship detection.

1. For wakes detected in boundary region $S_{3}$ and cloud neighborhood $S_{2}$, we calculate the length of the wake fitting curve belonging to the boundary region and cloud region, and the confidence of ship detection is given as

(23) $$P_{1}=QI\cdot \left(1-\prod_{i:l_{i}\geq l_{0}}\left(1-p_{i}\right)\right)$$

where $p_{i}$ is the goodness of fit according to the degree-of-freedom adjusted coefficient of determination; $l_{0}$ is the given length threshold.

2. For ship body neighborhood $S_{1}$, we first take the ship region in the detection box, and we use PCA to obtain the axis of the ship body, as described in Equation (12). We search for ship wakes in the front and back regions along the ship axis, respectively. Choosing the wake region with greater confidence, let $C_{0}$ denote the center of the wake region, and $C_{1}$ represents the center of the ship body. Then, the sailing direction ${\vec{l}}_{a}$ is obtained by

(24) $$\begin{array}{l} {\vec{l}}_{0}=C_{1}-C_{0},{\vec{l}}_{a}=y_{0}v_{2}\\ y_{0}=\underset{y_{0}\in \{1,-1\}}{\arg\min}\left(\frac{y_{0}v_{2}\cdot {\vec{l}}_{0}}{\left\|v_{2}\right\|\left\|{\vec{l}}_{0}\right\|}\right) \end{array}$$

Due to the influence of the quality of the image, it may not be possible to extract the correct recognition region in some cases. Therefore, we propose the following heuristic search algorithm to extract the ship body and wake regions for images with a large range of quality levels.

Step 1: Initialize the recognition threshold $b=b_{0}$.

Step 2: According to the output of extraction, the recognition region is divided and morphological calculation is performed.

Step 3: The threshold value is updated with the morphological calculation results.

Step 4: Repeat the calculation with the new threshold until the morphological calculation results meet the requirements for region extraction.

The initial search value of the threshold in the algorithm uses the following fuzzy algorithm based on a membership function:

(25) $$b_{0}=\theta^{T}\frac{\mu_{A}(w^{T}{\bar{x}}_{i})}{1^{T}\cdot \mu_{A}(w^{T}{\bar{x}}_{i})}$$

where $\theta \in R^{m}$ is the fuzzy set composed of reference thresholds under different image quality levels; m is the number of reference sample images used; and μA(wTx¯i)=[μA1(wTx¯i),…,μAm(wTx¯i)] is the corresponding membership function vector. The membership function adopts the following form of a Gaussian and exponential function:

(26) μA1(wTx¯i)=1/(1+ea1(wTx¯i−b1)),μAm(wTx¯i)=1/(1+e−am(wTx¯i−bm))μAk(wTx¯i)=e−ak(wTx¯i−bk)2,k=2,…,m−1

where $a_{i},b_{i}$ are the shape parameters of the corresponding membership function.

The threshold update process is as follows:

(27) $$\begin{array}{l} \Delta b=\pm \frac{\mathrm{sgn}(A_{1}-A_{0})}{1+e^{-a\left|A_{1}-A_{0}\right|}}\\ b^{\prime }=b-k\Delta b \end{array}$$

where $b,b^{\prime }$ are the thresholds before and after one update; $A_{1},A_{0}$ are the areas extracted in the morphological analysis and the area of the mean ship body and wake regions calculated according to the image resolution; sgn() is a sign function. The selection of the initial symbol is determined by the relationship between the threshold value under the criterion and the area change of the region. The principle is that a positive Δb will lead to a positive area change ΔA. $a\in R^{+}$ is a constant coefficient, and k is the basic search step, which, in this case, is 0.1.

## 4. Results and Discussion

### 4.1. Implementation Details

We evaluate the method described in the above section and demonstrate it on the AIR-MOT dataset [53]. This dataset is a large-scale and high-resolution Jilin1 satellite video dataset for multi-object tracking. We filter over 1000 images with sailing ships and 200 negative images with no ships in the ocean. The spatial resolution of these images is from 0.91 m to 1.27 m, the size of images is 1920 × 1080, and the sensor is an MSS. We use these data to evaluate our method. The AIR-MOT dataset is proposed for object tracking, and successive observations can help to obtain the true ship position even when the targets are invisible in a certain image. In the training process, the dataset is split into training and validation sets with an 80/20 split, the YOLOv5 network is used as the model backbone, and other hyperparameters, such as the DBSCAN parameters, are empirical constants. We use the detection accuracy and false alarm rate to evaluate the performance.

### 4.2. Sea Region Separation

**Figure 7:**
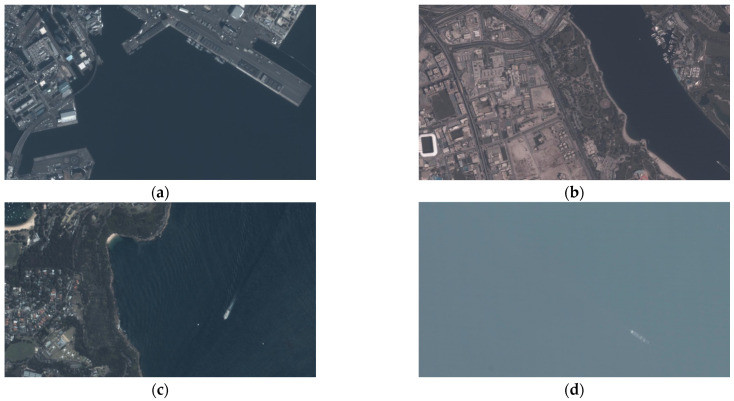
The original remote sensing images (**a**–**d**).

**Figure 8:**
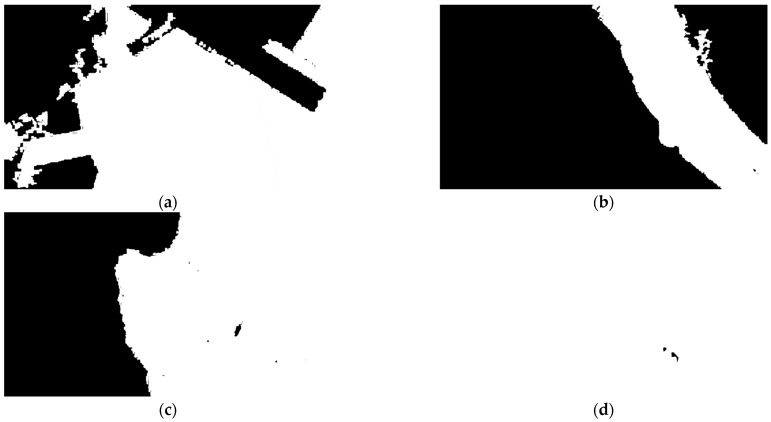
The performance of sea region separation (**a**–**d**).

We demonstrate the original remote sensing images in Figure 7. The corresponding sea region separation result are as shown in Figure 8, where the white pixels are the extracted sea region. Although these images have differences in resolution, hue, and definition, the presented method can effectively extract the sea region, with an Intersection of Union (IoU) of over 95%, within 1.1 s in the MATLAB environment. This result indicates that this method can achieve effective, adaptive, and fast sea region separation, which can be used to limit the detection region.

**Figure 9:**
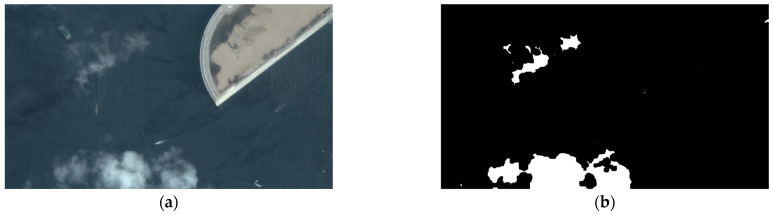
The performance of cloud detection (**a**,**b**).

The performance of cloud detection is shown in Figure 9: the original remote sensing image is shown in Figure 9a, and the cloud detection result is shown in Figure 9b, where the white pixels are the extracted cloud area. The average computing time is about 2.90 s. We can see that the detection operation can provide appropriate segmentation for further processing.

After sea area separation and cloud detection, we can obtain the sea background, the cloud neighborhood $S_{2}$, and the boundary region $S_{3}$.

### 4.3. Sea Background Analysis

**Figure 10:**
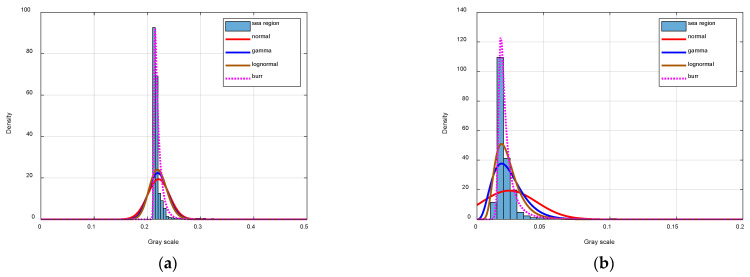
The modeling performance in the original grayscale (**a**) and standardized grayscale (**b**).

The performance of a normal distribution, the lognormal distribution [54], the Gamma distribution [55], and the Burr type XII distribution is compared. As shown in Figure 10, we first demonstrate the modeling performance in the original grayscale (Figure 10a) and standardized grayscale (Figure 10b). We can see that standardization is necessary in modeling the sea background.

**Figure 11:**
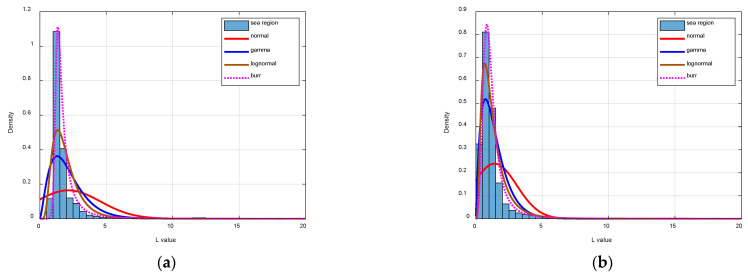

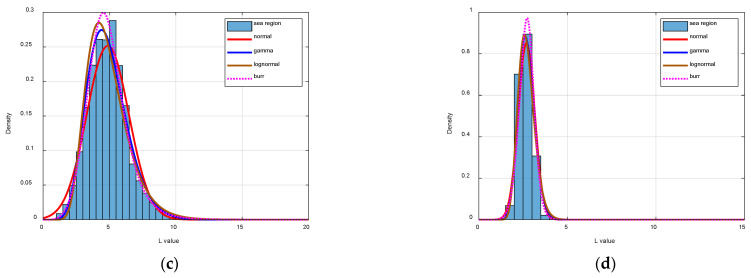
The performance of sea background modeling (**a**–**d**).

We model the sea background of the images shown in Figure 7, and the fitting distribution curves of the four models and the corresponding L-value histograms are shown in Figure 11. Visually, the Burr type XII distribution has the best performance in these four methods. For further evaluation, we calculate the Kullback–Leibler divergence (KLD) between the true distribution and modeling distribution (shown in Table 1). As we can see, the Burr type XII distribution has the lowest average and variance KLD regarding the true distribution, which indicates the Burr type XII distribution has the best modeling performance. Moreover, we find that the Gamma distribution tends to have lower KLD when the image has better quality, and this is the reason that we choose the Gamma distribution as the alternative distribution.

**Figure 12:**
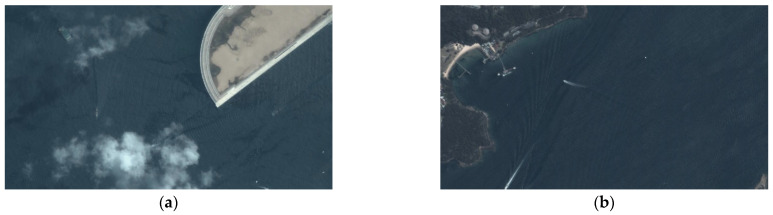
Challenging remote sensing images (**a**,**b**).

We present the image entropy $H_{e}$, the Tenengrad gradient $H_{T}$, the Gabor feature $H_{G}$, the reblur performance $H_{R}$, and the proposed criterion QI to evaluate the image quality in Figure 7 and Figure 12. Among these images, those in Figure 7c,d and Figure 12a,b are more conducive to the extraction of the ship wakes, and the image in Figure 7d provides the worst conditions. By comparing the quantitative results between the different methods in Table 2, we find that the proposed quality criterion can effectively evaluate the image quality, and it the has the best conformity in terms of intuition and practical operation.

### 4.4. Wake Analysis

**Figure 13:**
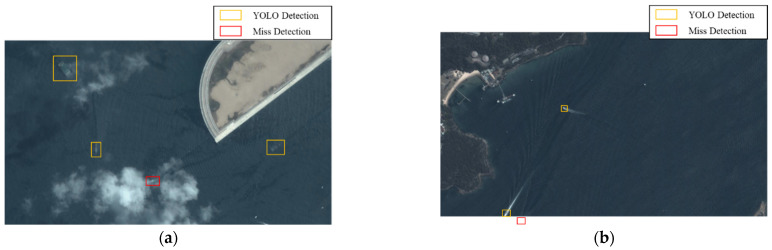
The YOLO detection results (**a**,**b**).

Our ship body detection has similar performance to that presented in [56]. In this paper, we only show the wake analysis results and the improvement. Figure 12 shows representative remote sensing images. The ship detection task is challenging in these images since the cloud coverage and the detection around the image boundary render certain ship targets almost invisible. Figure 13 shows the detection results for the YOLO network; the orange and red bounding boxes represent the YOLO detection result and missed detection target, respectively. This demonstrates that direct ship detection is infeasible in these cases.

**Figure 14:**
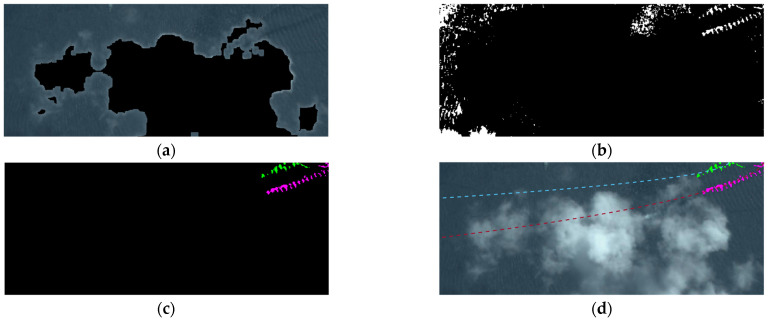
Wake detection for cloud neighborhood (**a**–**d**), where different colors represent different wake regions and their fitting curves.

We demonstrate the wake detection result of in Figure 12a and Figure 14. Figure 14a shows the searching region of the cloud neighborhood; here, the black pixels are the marked cloud pixels. Figure 14b shows the wake extracted using the method presented in Equation (17). We can see that many noise waves are also extracted. Figure 14c shows the ship wakes after clustering and checking; we find that the proposed method can effectively detect the wakes from the noisy environment. Figure 14d shows the wake fitting result. The goodness of fit is 0.6016 and 0.9320, respectively, and the calculated confidence of the ships in the analyzed cloud region is 0.9622. These results indicate that our method can help to detect ships covered by clouds in a remote sensing image.

**Figure 15:**
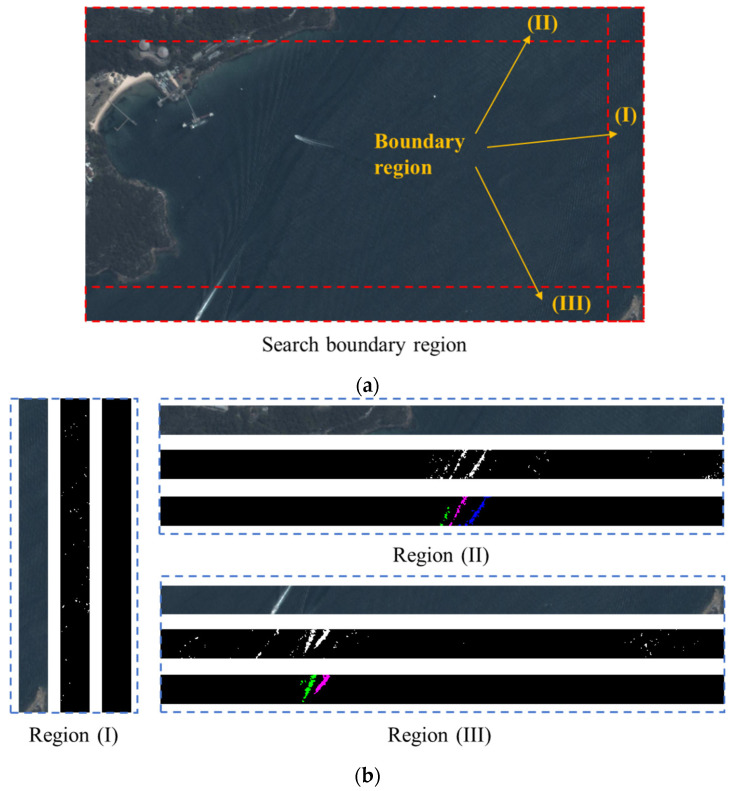
Wake detection around the image boundary (**a**,**b**).

Next, we demonstrate the performance of our wake detection method around the image boundary in Figure 15. Figure 15a shows the boundary regions of Figure 12b; according to the sea region analysis, only three boundary regions are needed to detect ship wakes. Figure 15b shows the detection results in the regions in Figure 15a, where we successively present the original boundary region, the binary boundary region, and the extracted wake regions. From these results, we observe that our method can effectively detect the ship wakes around the image boundary, and the calculated confidence of the ships in the analyzed boundary region is 0.9516 and 0.9438. Since no ship body detections are matched with the detected ship wakes, we can draw the conclusion that there are possible ships around boundary regions (II) and (III).

The statistical results in terms of the accuracy, false alarm rate, and GFLOPs of the detection network are shown in Table 3. Here, ${Acc}_{1}$ is the detection accuracy in the cloud cover region, ${Acc}_{2}$ is the detection accuracy in the image boundary region, ${Acc}_{av}$ is the general accuracy of ship detection based on wakes in the cloud cover region and image boundary region, FA is the false alarm rate in these cases, and GFLOPs is the number of floating-point operations of the detection network in billions.

The general accuracy of ship detection based on wakes in the cloud cover region and image boundary region is about 73%, with a false alarm rate of about 22%. In contrast, none of these ships can be detected by direct ship body detection, which means that the accuracy of ship detection is 0%. This result indicates that the proposed method is practical in ship detection.

**Figure 16:**
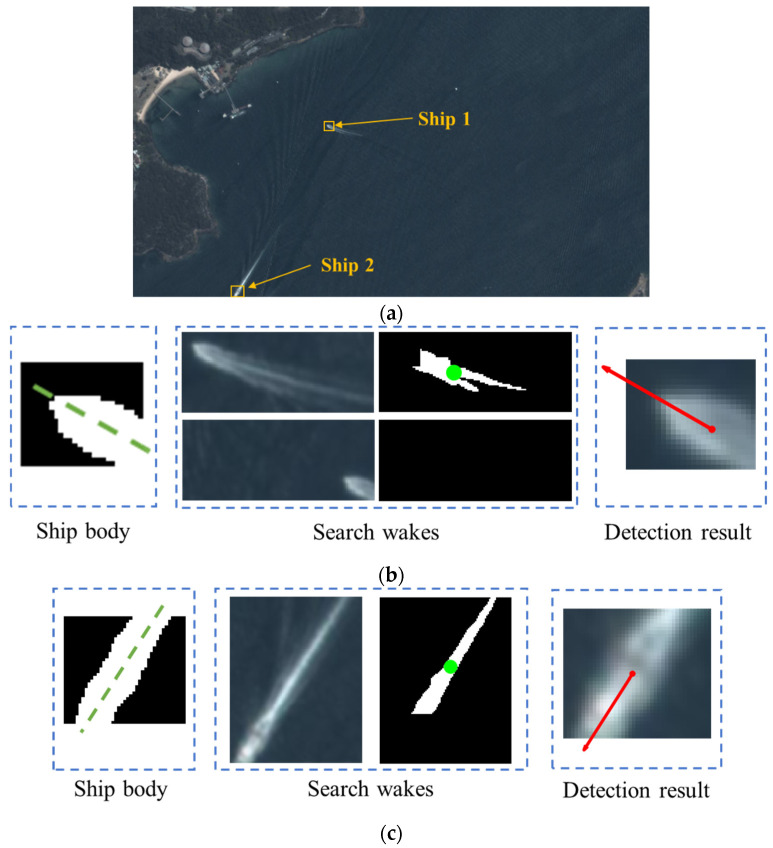
Wake detection for ship body neighborhood (**a**–**c**).

For ship body neighborhood $S_{1}$, the wake detection result is as shown in Figure 16. Figure 16a shows the YOLO detection results; for each detected ship, the wake searching progress is shown in Figure 16b,c. We can observe that, using the wake detection result, the accurate sailing direction is obtained and the confidence of ship detection is also improved. The direction error is about 1.3 deg and 1.6 deg for ship 1 and ship 2, respectively. This result shows that our method is effective for ship detection and in acquiring the ship state, which is helpful in analyzing and predicting the ship trace.

**Figure 17:**
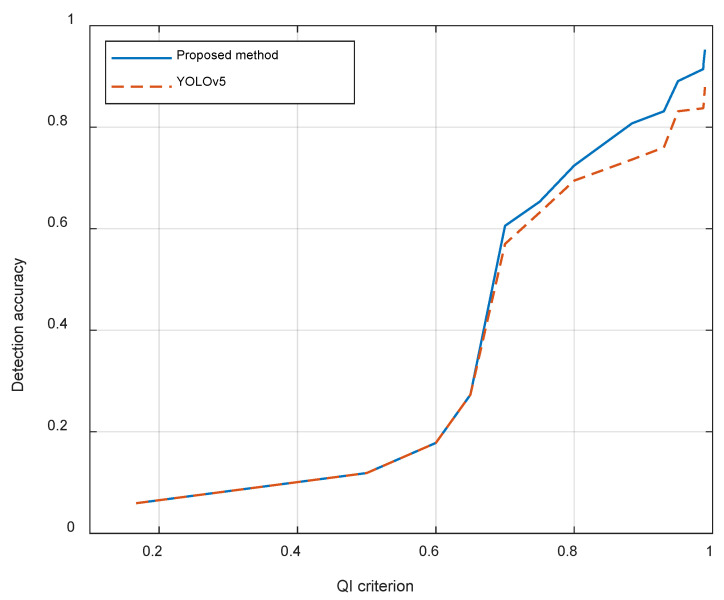
The change in the accuracy of detection when the the quality of the image *QI* changes.

The spatial resolution of these images ranges from 0.91 m to 1.27 m, but we do not have the specific spatial resolutions of these images. We present the method’s performance varying with the proposed criterion QI in Figure 17. Generally, a larger QI reflects a higher spatial resolution.

We can observe that the accuracy of detection increases when the QI increases, which indicates that the proposed method performs better in high-spatial-resolution and high-quality images. We can also observe that our method is only effective when QI>0.7, and it has a good improvement when QI>0.85. Since the wakes are faint in low-spatial-resolution or low-quality images, no additional information can be acquired using the proposed method.

**Figure 18:**
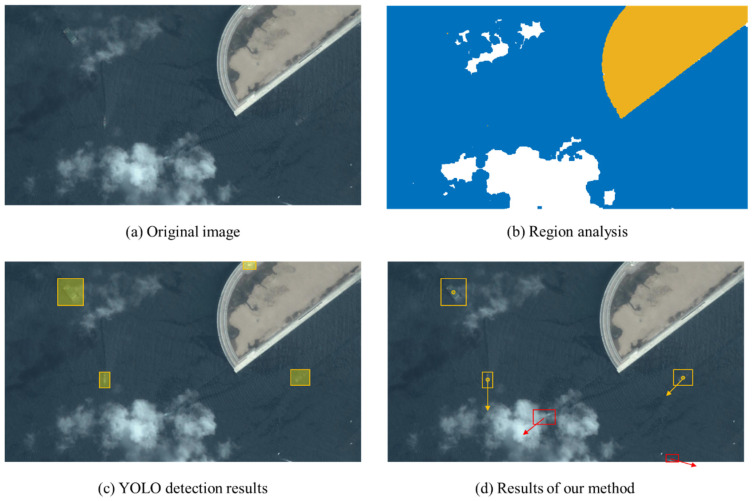
Detection results of our method.

An overview of the pipeline used in this method is shown in Figure 18. We can observe the advantages and improvements of our method.

The total process can be completed in 25 s. As shown in Figure 18, there are three visible vessels, one vessel covered by clouds, and one vessel outside the image. The YOLO detects all three visible vessels with one false alarm, and the general accuracy for visible vessels and invisible vessels is 75% and 0%. Using our framework, false alarms are eliminated and invisible vessels are also detected; the general accuracy for visible vessels and invisible vessels is 100% and 100%, and 80% of the vessel course can be acquired. From these results, we can conclude that our method has the following advantages:

1. The YOLO detection results can be checked, and some possible errors can be effectively eliminated;
2. The detection gaps caused by the cloud coverage and out-of-boundary objects can be filled with no additional sensors required;
3. The course of the sailing vessel can be acquired when checking the ship body detection at the same time;
4. No additional dataset is required for the presented wake detection method;
5. The boundary of the proposed method is also presented by briefly evaluating the image quality.

## 5. Conclusions

This paper presents a practical and effective scheme for ship detection in visible remote sensing images. The method combines the advantages of deep convolutional neural networks and feature-based image processing. We fuse ship body detection and ship wake detection in a real application, and the detection gaps caused by the cloud coverage and out-of-boundary targets can be filled with no additional sensors required. Our method uses the YOLOv5 network to achieve the fast and effective detection of the ship body; the wake detection method is used according to different types of regions. A sea region analysis method is proposed to judge the state of the sea region and the quality of the sea region. We propose a robust ship detection method using wakes for challenging cloud cover and image boundary regions, where ships are usually invisible in a remote sensing image. The extracted sea region is modeled using the Burr type XII distribution, and the ship wakes are detected using the distribution of the modeled sea region. Practical engineering problems such as measurement limits, result examination, and time efficiency are also considered in our method for real applications. The results show that our method can achieve over 70% accuracy even when the ship body is invisible in a remote sensing image, and accurate sailing detection can also be obtained.

The presented study also has certain limitations. First, the proposed method is based on the YOLOv5 network, and only the false detections due to cloud cover and the image boundary region can be corrected. Second, the accuracy of wake detection can be further improved. Moreover, the detection performance is partly influenced by the quality of the images, and the noise resistance capability can also be further improved.

In the future, we will attempt to use more effective ship detection methods to improve the detection accuracy and robustness. We will also study the wake detection method on lower-quality imagery.

## Figures and Tables

**Table 1:** KLD of different methods.

	Normal	Gamma	Lognormal	Burr Type XII
Image (a)	2.2350	0.9067	0.4849	0.2664
Image (b)	1.2977	0.1998	0.1340	0.2028
Image (c)	0.0437	0.0250	0.0753	0.0157
Image (d)	0.0697	0.0439	0.0513	0.0561

**Table 2:** Performance of image quality indicators.

	$H_{e}$	$H_{T}$	$H_{G}$	$H_{R}$	QI	Intuition
Figure 7a	2.3050	21.5425	4.7419	0.9841	0.8836	common
Figure 7b	2.5348	12.9861	5.6017	0.9679	0.9296	common
Figure 7c	3.8685	50.5564	7.3810	0.9660	0.9870	good
Figure 7d	2.1113	2.9499	5.4744	0.9971	0.1669	bad
Figure 11a	5.4253	90.8874	7.2396	0.9701	0.9890	good
Figure 11b	3.7863	79.6189	7.3615	0.9607	0.9865	good

**Table 3:** Performance of detection.

Method	${Acc}_{1}$	${Acc}_{2}$	${Acc}_{av}$	FA	GFLOPs
YOLOv5	0%	0%	0%	-	135.0
YOLOv8	2%	0%	1%	-	165.2
YOLOX	2.5%	0%	1.25%	-	185.3
Our method (YOLOv5-based)	75%	72%	73%	22%	135.0

## Data Availability

The datasets generated and/or analyzed during the current study are available in the AIR-MOT repository, at https://github.com/HeQibin/TGraM (accessed on 1 September 2023), and can be found in the published article [53]. Q. He, X. Sun, Z. Yan, B. Li and K. Fu, “Multi-Object Tracking in Satellite Videos with Graph-Based Multitask Modeling”, in IEEE Transactions on Geoscience and Remote Sensing, vol. 60, pp. 1–13, 2022.
